# Antioxidant Effects of Catechins (EGCG), Andrographolide, and Curcuminoids Compounds for Skin Protection, Cosmetics, and Dermatological Uses: An Update

**DOI:** 10.3390/antiox12071317

**Published:** 2023-06-21

**Authors:** Gatien Messire, Raphaël Serreau, Sabine Berteina-Raboin

**Affiliations:** 1 Institut de Chimie Organique et Analytique ICOA, Université d’Orléans-Pôle de Chimie, UMR CNRS 7311, Rue de Chartres-BP 6759, 45067 Orléans CEDEX 02, France; gatien.messire@univ-orleans.fr; 2 Unité de Recherche PSYCOMADD, APHP Université Paris Saclay, Hôpital Paul-Brousse, 12 Avenue Paul Vaillant Couturier, 94804 Villejuif, France; raphael.serreau@epsm-loiret.fr; 3 Addictologie EPSM Georges DAUMEZON, GHT Loiret, 1 Route de Chanteau, 45400 Fleury les Aubrais, France

**Keywords:** green tea catechins (ECGC), andrographolide, curcumin, skin photoprotection, skin permeation, oxidative stress, antimicrobial, cosmetic and dermatologic formulations

## Abstract

Here we have chosen to highlight the main natural molecules extracted from *Camellia sinensis*, *Andrographis paniculata*, and *Curcuma longa* that may possess antioxidant activities of interest for skin protection. The molecules involved in the antioxidant process are, respectively, catechins derivatives, in particular, EGCG, andrographolide, and its derivatives, as well as various curcuminoids. These plants are generally used as beverages for *Camellia sinensis* (tea tree), as dietary supplements, or as spices. The molecules they contain are known for their diverse therapeutic activities, including anti-inflammatory, antimicrobial, anti-cancer, antidiabetic, and dermatological treatment. Their common antioxidant activities and therapeutic applications are widely documented, but their use in cosmetics is more recent. We will see that the use of pharmacomodulated derivatives, the addition of co-antioxidants, and the use of various formulations enable better skin penetration and greater ingredient stability. In this review, we will endeavor to compile the cosmetic uses of these natural molecules of interest and the various structural modulations reported with the aim of improving their bioavailability as well as establishing their different mechanisms of action.

## 1. Introduction

The skin, which undergoes natural wear and tear, is the largest human organ, and protecting it against various aggressions is a public health issue. Skin aging has two origins [1,2]. The first (intrinsic aging) is due to problems in the meshwork of collagen and elastin fibers, while the second (extrinsic aging) stems from environmental factors such as solar radiation. Oxidative stress plays an important role in inducing inflammation [3,4], which limits epidermal cell renewal and ultimately leads to a reduction in epidermal thickness, weakening the protective barrier [5]. These radiations induce the production of reactive oxygen radicals (ROS), which affect keratinocytes, important cells in the epidermis. In response to this aggression, keratinocytes produce pro-inflammatory cytokines such as interleukin-8 (IL-8) and tumor necrosis factor-α (TNF-α) [6,7], which in a vicious circle produce even more ROS [8]. Hyaluronic acid (HA), a well-known component of the skin matrix, is present in two layers: the dermis and the epidermis. However, as skin aging and epidermal degradation lead to skin dehydration [9], it is also necessary to promote hydration. The addition of antioxidants is essential to prevent the oxidation of cosmetics, medicines, and, more generally, foodstuffs and ensure their preservation. Synthetic antioxidants have often been used in various fields of application, but when it comes to cosmetics, it is preferable to use natural products, whether derived from plants or possibly marine organisms. Well-known natural antioxidants include certain vitamins and various molecules from plants commonly used as food or dietary supplements [10]. Some of these plants also have antibacterial or anti-inflammatory activities, which may be of interest for the safety of formulations or health, as many cancers start with inflammation that becomes chronic [11]. 

Although there are many plants with antioxidant activity, in this review, we focus on the various products extracted from the plants *Camellia sinensis* (L.) Kuntze (green tea), *Andrographis paniculata* (Burm. f.) Wall ex Nees, and *Curcuma longa* (L.), (turmeric), which can be used for dermatological purposes, protecting the skin against aging, against solar UV radiation responsible for cutaneous carcinoma, but also against the cutaneous aggression of anti-cancer treatments and other oxidative stresses [12,13,14,15]. 

These three plants were chosen both for their common activity and for our long-standing interest in them. Within the team, we first became interested in *Andrographis paniculata* for its anti-inflammatory activity, then more recently in *Curcuma longa* and *Camellia sinensis*. With regard to turmeric, in view of the few publications questioning its efficacy, we are trying to verify its anti-inflammatory and antioxidant activities in vitro and then in vivo, but we are only at the beginning. As for *Camellia sinensis*, already widely studied, it caught our attention because its activity has been proven, but its instability and bioavailability problems limit its use. It was, therefore, necessary to review the literature on these three plants, widely used as beverages or dietary supplements, in order to consider the best tests or pharmacomodulations to implement to potentiate activity and/or bioavailability for cosmetic or dermatological applications. The bibliographic search was carried out using the following keywords: [*Camellia sinensis* skin effects] and [antioxidant], which yielded 40 publications, then [EGCG skin effects] and [antioxidant] and [cosmetics] which yielded 88 publications with overlaps with the previous search. For *Andrographolide*, the bibliographic search was carried out using the keywords [andrographolide skin effects] and [cosmetics], resulting in 26 publications. For *Curcuma longa*, the keywords were [curcumin skin effects] and [cosmetics], which gave rise to just 7 publications, mainly patents.

The various active compounds present in these three plants will be detailed. Their major drawback remains their low bioavailability and poor skin penetration, which is why the use of their extracts in cosmetic products has so far been limited despite their interest. For these reasons, we will also focus on the structural modifications or various carriers that have been attempted to overcome this problem.

With regards to the compounds present in *Camellia sinensis,* in the Theaceae family, we will be interested mainly in epicatechin gallate (EGCG) and the differentiations achieved either by glycosyl substitution of certain alcohol functions of these polyphenols [16,17,18] or by encapsulation [19] or by attachment to nanoparticles [20,21,22,23]. These antioxidants are health products, as they are also used against diseases such as neurological disorders [24], obesity disorders [25], cardiovascular pathologies [26], and of course, anti-aging without being exhaustive in their possible application. The antioxidant [27], anti-microbial [28], anti-inflammatory [29], and anti-carcinogenic [30,31,32,33] activities of the polyphenols present in green tea have been widely reported [34,35]. The efficacy of catechins is correlated with various factors, such as the accessibility and position of the functional groups, mainly hydroxyls, as well as their stability or level in the various extracts. 

In Chinese or Ayurvedic pharmacopeia, *Andrographis paniculata* [36] was used to treat pulmonary infections. This plant of the Acanthaceae family contains several bioactive compounds, the best known of which is a labdane diterpenoid called andrographolide. These compounds have a wide range of biological activities, including anti-inflammatory [37,38,39], antipyretic [40,41], hepatoprotective [42,43,44,45], anti-thrombotic [46,47], immunostimulant [48,49], anti-viral [50,51,52], antioxidant [11,53], and anti-cancer [54,55]. We have already reported on the antioxidant mechanisms and the regulation of the Nrf2 (Nuclear factor (erythroid-derived 2)-like 2) signaling pathway by andrographolide that interested us here [53]. These compounds could be of interest in cosmetic formulations.

Curcuminoids are the main constituents of turmeric (*Curcuma longa* (L.), Zingiberaceae family). One of them, curcumin, is well known for its anti-inflammatory [56], antioxidant [56], anticarcinogenic, chemopreventive [57], and anti-tumor [58] effects when taken alone or in combination with other plant extracts such as ginger. Ginger belongs to the same Zingiberaceae family. The anti-cancer effects of its compounds are thought to be due to their ability to inhibit cell proliferation and ROS induction. Curcumin also acts as a free radical scavenger and exhibits antioxidant activities, with enhanced superoxide dismutase (SOD) activity, and also declined malondialdehyde (MDA) and lipofuscin levels [57], thanks to its phenolic parts, and has more recently been used in cosmetic formulations [59,60,61,62,63].

Although the molecules present in these three plants have varied therapeutic interests, in this review, we will focus on cosmetic or dermatological applications.

## 2. Results and Discussion

### 2.1. Camellia sinensis (Grenn Tea)

*Camellia sinensis* is of considerable interest for its health benefits, as its extracts contain a variety of catechin polyphenols or flavan-3-ols, which account for over 30% of the weight of dried leaves [64,65] and are responsible for green tea’s activities. Although epigallocatechin-3-gallate (EGCG) is the most abundant catechin (around 65% of total catechins), green tea also contains significant amounts of other catechins: epicatechin, epicatechin-3-gallate, and epigallocatechin [65] (Table 1). The quantities and levels of polyphenols depend on the tea variety, growing conditions (i.e., the environment), drying conditions, and extraction process [66]. We are particularly interested in green tea, although it accounts for only 20% of production; as fermentation processes are more advanced for black tea, a certain number of polyphenols could be damaged, which would explain the lower therapeutic activity of aqueous extracts.

#### 2.1.1. Extracts in Cosmetics or Pharmaceutical Formulations

Extraction solvents currently used are water or a mixture of water with other polar organic solvents such as methanol, ethanol, or acetone. Organic solvents allow better extraction but are difficult to use in cosmetic companies. Lee et al. [67] used deep eutectic solvents; these mixtures replacing ionic liquids were reported to have low toxicity and very good biodegradability while solubilizing a large number of structures, making these deep eutectic solvents good extraction solvents. They developed BGG-4 composed of betaine, glycerol, and D-(+)-glucose in the following proportions 4/20/1 and were able to extract catechins from tea very efficiently. In addition, these solvents are compatible with the use of extracts in cosmetics or pharmaceutical formulations. It would be interesting to extract green tea leaves in green organic solvents such as, for example, limonene or the green solvent eucalyptol recently highlighted by our team [68,69].

#### 2.1.2. Pharmacomodulations and Biological Activities

Polyphenols can also undergo rapid degradation, and pharmacomodulations have been carried out to increase the stability and bioavailability of these antioxidants. Although these alkylated gallate esters (Table 1) showed very good antioxidant properties and increased bioavailability, some of them also showed cytotoxicity in rats [28,65]. 

The antioxidant activity of catechins is thought to be due, on the one hand, to regulation of the Nrf2 (Nuclear factor (erythroid-derived 2)-like 2) signaling pathway. Secondly, catechins are thought to stimulate the NF-kB and MAPK pathways, thus balancing cellular redox status. Despite the interest in these compounds, they are still little used in the cosmetics industry. This may be due to the difficult permeability of the skin, resulting partly from the chemical nature of catechins, which can interact with skin lipids, and partly from their hydrophilic nature due to numerous hydroxyls. To overcome the lack of penetration of EGCG and ECG compared with EC and EGC, some researchers have considered formulations such as emulsions, ointments, transdermal patches, liposomes, or microparticles and nanoparticles. Waranuch et al. [70] showed that chitosan microparticles (<5 mm) loaded with green tea extracts offered better skin permeability, particularly for non-galloylated catechins such as EGC and EC, whereas galloylated analogs of EGCG and ECG did not penetrate as well. This is probably due to the encapsulation of the molecules, which limits their degradation under the action of cutaneous enzymes.

Skin hydration combats one of the intrinsic processes of skin aging, hyaluronic acid, which is involved in the regulation of hyaluronic acid synthase (HAS). Cho et al. [71] studied the mechanisms of action of EGCG on skin hydration by measuring HAS and HYAL (hyaluronidase) enzymes using cell proliferation assays. They showed that HYAL expression levels decreased under UV irradiation of HaCaT cells. In addition to its anti-oxidant activity, EGCG is also thought to reduce melanin secretion, and may therefore have a skin-lightening effect. All this makes EGCG a potential cosmetic ingredient in many respects. In extracts of green and oolong teas, which represent less than 2% of teas consumed, a methylated form of EGCG, (−)-epigallocatechin-3-(3″-*O*-methyl) gallate (3″ Me-EGCG) (Table 1), has been found in small quantities. Its activity is not yet well known, and Cho et al. [72] evaluated the antioxidant properties of keratinocytes (HaCaT cells) using different analytical systems. (3″Me-EGCG) showed increased expression of heme oxygenase 1 (HO-1), protecting keratinocytes by regulating the survival protein AKT1 in HaCaT cells. Protein kinase B (AKT) participates in the PI3K/AKT pathway, which induces cell survival by acting on cell proliferation and survival. 3″Me-EGCG, therefore, also possesses antioxidant properties desired for a cosmetic product. 

#### 2.1.3. Cosmetic Formulations for Improved Bioavailability

The limited availability of these agents can be overcome by working on cosmetic formulations of the emulsion [73], encapsulation, and micro or nanoparticles type, which allow excellent penetration of all skin layers. To improve penetration, EGCG was formulated by Boncu et al. [74] in controlled-release systems for anti-aging cosmetic applications using ethosome-based formulations. These were prepared by mechanical dispersion with a gelling agent, Carbopol 980. Ethosomes also have the advantage of protecting encapsulated compounds from various environmental factors [75]. Particle size is a few hundred nanometers, around 200 nm in this case. In this paper, six ethosome formulations were developed, and in vitro encapsulation and release efficiencies were investigated. The authors showed that these formulations were non-toxic, had a good percentage of encapsulation efficiency, and penetrated the skin well, comparing the results with oral administration. The ethosomal formulations also showed good organoleptic properties; it is conceivable that these formulations are currently under development in the cosmetics industry. A similar study was also carried out in 2022 on niosome-loaded EGCG, a drug-transport system based on a non-ionic surfactant and cholesterol in this case [22]. This system improves dermal penetration of various drugs for cutaneous application and can be used for therapeutic or cosmetic bioactive compounds. It should be noted that this type of encapsulation has also been used for curcumin or rutin [20,76]. Peterson et al. [77] have used lipid nanoparticles to load EGCG, vitamin E, and resveratrol carriers for cutaneous and transdermal drug delivery. Solid and lipid nanoparticles have properties similar to those of liposomes or emulsions. Like ethosomes, nanoparticles are stable; they have good encapsulation capacity, protect the encapsulated molecules, and enable good release of the active molecule. In this article, unfortunately, EGCG was not protected from UV-induced degradation by nanoparticles, whereas vitamin E and resveratrol were.

#### 2.1.4. Synergistic Effects of Adding Other Antioxidants

With EGCG being photosensitive, Bianchi et al. [78] tested the addition of various antioxidants such as vitamin E, butylated hydroxy-toluene (BHT), vitamin C, and α-lipoic acid to cosmetic creams. The photodegradation of these creams containing EGCG with varying proportions of the four co-antioxidants tested was measured by HPLC. This showed that the EGCG degradation was considerably attenuated by vitamin C but increased by vitamin E and that α-lipoic acid had a very good stabilizing effect, while BHT had no effect.

In conclusion, epigallocatechin-3-gallate (EGCG) has several dermatological applications. It has been shown to interact with hyaluronic acid, enhancing its antioxidant activities. It is a good ingredient in sun creams and for combating skin diseases such as psoriasis, alopecia, dermatitis, atopic eczema, and skin cancer, but its instability as a function of pH values or UV irradiation reduces its performance [79]. As explained above, this instability can be limited by the use of certain formulations or the addition of other antioxidant ingredients, or by the loading of EGCG onto titanium dioxide. EGCG has many very interesting properties, and some formulations are more promising than others in vitro, however, more targeted studies should be carried out with different formulations and constant EGCG contents. It is prudent not to use tea extracts marketed for cosmetic or other purposes, with the exception of pharmacopeia extracts. The EGCG contents of these extracts are varied. Poorly preserved, their content may further decrease due to the instability of EGCG, and their composition may vary [80].

### 2.2. Andrographis Paniculata

#### 2.2.1. Composition and Distribution within the Organism

Several studies have been carried out on this plant to monitor extracts in different solvents, and twenty-seven ent-labdane diterpenoids derivatives and fourteen glycosylated or non-glycosylated flavonoids are referenced and described [36]. The compounds mainly found in the extracts are listed in Table 2, among which are diterpene labdanes deoxyandrographolide, andrographolide, 14-deoxy-11,12-didehydroandrographolide, and neoandrographolide diterpene glucoside. Lii [81] reported that andrographolide, like most phytochemicals, was rapidly metabolized and excreted in rats.

On repeated oral administration of andrographolide in rats, it was observed that andrographolide was stored mainly in the kidneys and liver before being found in the brain. If this molecule crosses the brain barrier, it should be usable in cosmetic and/or dermatological formulations [82]. Andrographolide is soluble in polar solvents such as acetone, alcohols, and ethers but insoluble in water, which limits its therapeutic use [83]. As for EGCG, the use of vectors such as microparticles, microemulsions, and nanocarriers is of interest for its formulation [84]. Their efficacy is such that Wang et al. [85] demonstrated that the bioavailability of andrographolide was increased by 241% by nanoparticles compared with andrographolide suspension.

#### 2.2.2. Biological Activities and Antioxidant Potential

Andrographolide neutralizes free radicals, protects mitochondrial integrity by activating pro-oxidant and/or antioxidant enzymes, and regulates the transcription factor Nrf2, which is involved in antioxidant defenses [86]. Sies et al. [87] showed that andrographolide had the ability to scavenge a stable free radical, 1,1-diphenyl-2-picrylhydrazyl (DPPH), more significantly than other known antioxidants such as ascorbic acid or BHT (butylated hydroxytoluene). Andrographolide had the highest antioxidant properties with the lowest IC50 value: 3.2 mg/mL compared to ascorbic acid and BHT. Several studies have been carried out on aqueous or alcoholic extracts of *Andrographis paniculata* showing ROS scavenging, which may be explained by the presence of flavonoids and phenolic compounds in the extracts. This inhibition of the formation of free radicals such as superoxides, hydroxyl radicals, and nitric oxide and the inhibition of lipid peroxidation by the extracts has been demonstrated in vitro and in vivo [88,89,90]. More surprisingly, this activity is also observed with pure andrographolide, which has no phenolic part in its skeleton. We have studied the antioxidant power of the main compounds in a methanolic extract made from commercially available dietary supplements [39]. We investigated the anti-aging properties of methanol extract, andrographolide, neoandrographolide, 14-deoxyandrographolide, and 14-deoxy-11,12-didehydroandrographolide in human keratinocytes. The methanolic extract was analyzed by HPLC and found to contain 0.87% andrographolide, which is not negligible, with the other compounds being present in lesser proportions [53]. We were also able to purify and isolate the main constituents of this extract by column chromatography [39] but did not identify any flavonoids. In this study, we demonstrated the beneficial effect of methanol extract against oxidative stress and inflammation in keratinocytes. Methanol extract decreased ROS production and TNF-α expression in HaCaT under pro-oxidative and pro-inflammatory conditions, respectively. We were able to show an antioxidant effect in keratinocytes of the methanolic extract at 5 μg/mL and of the compound 14-deoxyandrographolide at 1 μg/mL. Similarly, we demonstrated that methanolic extract and 14-deoxyandrographolide decreased ROS production in a primary culture of human dermal fibroblasts under conditions of oxidative stress [91]. In the same study, we were also able to show that methanolic extract at 5 µg/mL significantly reduced TNF-α expression under inflammatory conditions but not IL-8 secretion in HaCaT. The same reduction in TNF-α expression was observed in HDFa cells using methanolic extract (5 µg/mL) and pure commercially available andrographolide (5 µg/mL), but still no reduction in IL-8 secretion.

Superoxide dismutase (SOD), catalase (CAT), and glutathione peroxidase (GPx) represent the body’s enzymatic defenses against oxidative stress, whatever the source. Several studies have shown that andrographolide can restore SOD and CAT activities in cells subjected to oxidative stress [92]. It was shown on the skin of mice exposed to UV radiation that the application of andrographolide sodium bisulfate (0.4–1.2 and 3.6 mg/mouse) led to a dose-dependent increase in SOD and CAT activity [93]. Nrf2 (Nuclear factor (erythroid-derived 2)-like 2), already mentioned above, is an essential transcription factor that activates a wide variety of genes involved in antioxidant defense. Nrf2, therefore, plays an important protective role in balancing the oxidation-reduction reactions involved in oxidative stress by inducing the expression of specific enzymes. In vitro studies with andrographolide showed an increase in Nrf2 expression and nuclear translocation, irrespective of the cell type studied. This led to increased expression of the following enzymes with cytoprotective, antioxidant, and detoxifying effects: SOD, CAT, GCLC, GCLM, SRXN1, TXNRD1, GSR, GS, and GR. Andrographolide via the Nrf2 pathway increases the expression of the stress protein HO-1 which protects against oxidative attack [94,95,96,97]. In vivo, andrographolide also increases Nrf2 translocation and the activity of SOD, CAT, GSH reductase, and the antioxidant proteins SOD1, GST Ya, GST Yb, HO-1, GCLC, and GCLM. Studies have been carried out on rats and mice intraperitoneally [42,98,99].

Collagen decreases during intrinsic aging, while it increases during extrinsic aging. We have shown that the compound neoandrographolide at 5 μg/mL could decrease type I procollagen in HDFa [91]. Such a compound could therefore be of interest in reducing photoaging and hence disorganization of cutaneous connective tissues. Zhan et al. [93] reported that andrographolide sodium bisulfate (1.2 and 3.6 mg/mouse) could limit UV-induced collagen degradation in mouse skin. This makes it a plant of choice for cosmetic formulations. Ren et al. [100] also showed that andrographolide could have an effective action on acne, a chronic inflammatory follicular disease of the pilocytic units of the face. The presence of Propionibacterium acnes induces the production of pro-inflammatory cytokines. They demonstrated the inhibition of these pro-inflammatory cytokines (IL-8 and TNF-α) in acne without being able to elucidate the mechanism of action of andrographolide and other natural compounds on cytokine inhibition.

Among the many therapeutic applications of this plant is oncology, in particular skin cancer, which is on the increase and can spread if not treated effectively. Recently, Nagajyothi et al. [101] showed that andrographolide could be used for this type of cancer, which involves several complex mechanisms of inhibition or inactivation of signaling pathways with very limited toxicity. The authors listed its application on various skin cancer. Andrographolide is also said to have a skin-lightening effect. Fuongfuchat et al. [102] developed a nanoemulsion formulation of andrographolide for skin cancer, with very promising results on skin distribution. *Andrographis paniculata* is, therefore, a promising plant for the development of lightening, anti-aging agents, and other cosmetic applications. Few clinical trials have been conducted on patients suffering from various pathologies. In 2018, Fabbrocini et al. [103] evaluated the evolution of epidermal melasma in 40 Caucasian women after 6 months of application of a gel formulation containing andrographolide with glabridin and apolactoferrin. They reported 92.5% of good patient tolerance. Significant improvement was achieved with easy cosmetic use. These results need to be controlled by a placebo in a double-blind clinical trial.

### 2.3. Curcuma Longa

#### 2.3.1. Composition and Activities

**Figure 1:**
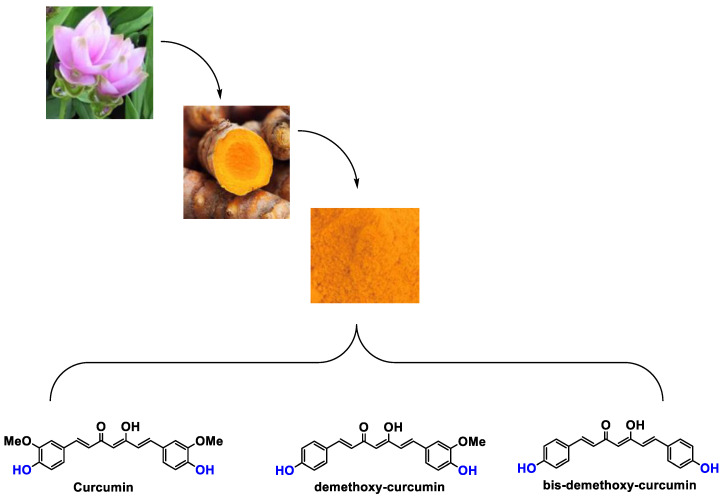
Various compounds found in turmeric; curcuminoid extract contains curcumin which makes up 60–70% by weight, demethoxycurcumin 20–27%, and bisdemethoxycurcumine 10–15%. Curcumin is not as the diketone but under enol form in solution.

It is now well established [104] that curcumin extracts or curcumin powder contain three main compounds and several others in much smaller quantities. These three compounds are (1E,6E)-1,7-bis(4-hydroxy-3-methoxyphenyl)-1,6-heptadiene-3,5-dione, present in 60−70% of the crude extract, demethoxycurcumin in 20–30%, and bisdemethoxycurcumin in 10% (Table 3 and Figure 1). A number of studies have been carried out on curcumin as a replacement for anti-inflammatory drugs (NSAID), which can have undesirable side effects such as renal damage [105,106], in order to find alternatives for the treatment of rheumatoid arthritis [107] or other inflammatory diseases. Du et al. [108] produced a synthetic pharmacomodulation of curcumin and showed anti-inflammatory activity. A large number of studies have been carried out with sometimes contradictory results, and in 2017 a review was published on the inventory of research and doubts about the effectiveness of curcuminoids [109]. Despite this, researchers continue to study this widely used spice. Its main problem lies in its instability in biological media in vitro and in vivo, which has led to disappointing clinical trials to date [110]. However, at the therapeutic level, some researchers have also described low potency, poor pharmacodynamics, and toxic effects [109,111]. 

#### 2.3.2. Cosmetic Use

With regard to the cosmetic aspect that interests us here, few in vivo biological studies have been reported. Dermatological and cosmetic preparations with effects for the treatment of skin diseases and the stimulation of hair growth have been developed from capsaicins, sinapins, or curcuminoids and are the subject of patents. For example, curcumin encapsulated in a high skin permeability protein nanoparticle used for hair application has been patented by Kazutaka [58]. Tetrahydrocurcuminoids (THC) are derived from the reduction of curcumin in vivo and are good antioxidants. They are, therefore, manufactured from curcumin in the presence of micro-organisms. THC has been tested and found to have anti-inflammatory and powerful antioxidant effects interesting for use in anti-aging cosmetics, skin-lightening products, and topical formulations. They prevent the skin from damage caused by infrared radiation, in particular. Curcuminoids and THC are dose-dependent, and THC is, surprisingly, more effective at lower concentrations for quenching free radicals. However, for other applications, it is curcumin that would have a higher activity than THC. The mechanisms of action of these compounds are different [62]. Curcuminoids are not the only antioxidant used in sun creams or moisturizers, as are N-acetylcysteine, certain vitamins, notably vitamin E (tocopherol), β-carotenes, resveratrol, and various other plant extracts. Curcumin is also used to improve dry skin problems leading to dermatitis as well as to limit wrinkles and ailments and treat skin disorders, such as dermatitis and itching [59,60,61,62]. Although numerous studies have been carried out on the therapeutic applications and anti-inflammatory activity of curcumin derivatives, its antioxidant and anti-aging activity is the subject of only a few patents but should develop in the coming decade on dermatological and cosmetic aspects, given the strong interest of scientists involved in the cosmetics field for natural products.

## 3. Conclusions

To sum up, in this review, we wanted to take stock of the literature and its conclusions on the cosmetic and dermatological interest of commonly available plants, two of which are widely consumed (Table 4). The three plants we are currently working on in our team are of particular interest to us because of their frequent use as food or dietary supplements and for the antioxidant compounds they contain. It, therefore, seems essential to us to persevere, on the one hand, in understanding their various mechanisms of action, and on the other, in studying possible synergies between the compounds present in the plant. This review of the literature, which does not reveal any particular toxicity of these compounds, enables us to envisage further research into the dosages needed to maximize efficacy and into the studies and potential pharmacomodulations that need to be carried out to improve skin penetration problems, particularly for dermatological uses. Similarly, solutions still need to be found to limit the instability of these various molecules. Given the problems of inflammation at the root of many pathologies, aging, particularly of the joints, and the side effects of many anti-inflammatory drugs (NSAIDs) currently on the market, andrographolide or curcumin could offer new therapeutic possibilities. Whether modified or not, these natural compounds could represent the drugs of the future for the prevention of cancers, particularly skin cancers, which are highly aggressive. In this review, green tea was chosen because EGCG is less degraded there than in fermented black tea, and more in-depth studies should also be carried out. Randomized placebo-controlled studies are needed to confirm or refute the real impact of these extracts in dermatological preparations.

## Figures and Tables

**Table 1:** Structure of main compounds found in *Camellia sinensis (Grenn tea)* and (−)-Epigallocatechin-3-(3″-O-methyl) gallate (3″-Me-EGCG).

Name	Structure and Details
(−)-Epicatechin (EC)	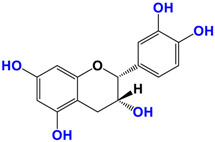 C_14_H_14_O_6_ MW: 278.26 g/mol
Catechin (C)	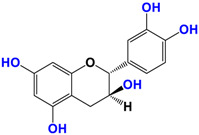 C_14_H_14_O_6_ MW: 278.26 g/mol
(−)-Epigallocatechin (EGC)	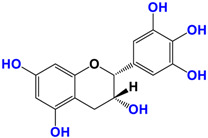 C_14_H_14_O_7_ MW: 294.26 g/mol
Gallocatechin (GC)	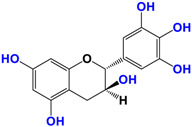 C_14_H_14_O_7_ MW: 294.26 g/mol
(−)-Epicatechin gallate (ECG)	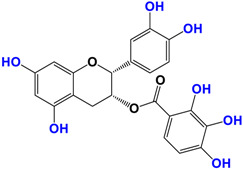 C_21_H_18_O_10_ MW: 430.36 g/mol
(−)-Epigallocatechin gallate (EGCG)	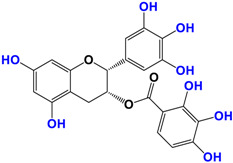 C_21_H_18_O_11_ MW: 446.36 g/mol
(−)-Epigallocatechin-3-(3″-O-methyl) gallate (3″-Me-EGCG)	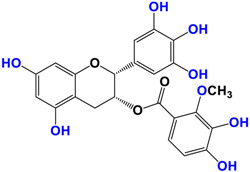 C_22_H_20_O_11_ MW: 460.39 g/mol
Alkyl gallate	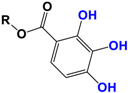

**Table 2:** Structure of main ent-labdane compounds found in *Andrographis paniculata*, andrographolide, neoandrographolide, 14-deoxyandrographolide, and 14-deoxy-11,12-didehydroandrographolide and two examples of flavonoids: 7,8-dimethoxy-2′-hydroxy-5-O-b-dlucopyranosyloxyflavone and Luteolin.

Name	Structure and Details
Andrographolide	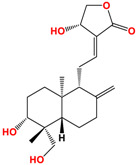 C_20_H_30_O_5_ MW: 350.45 g/mol
Neoandrographolide	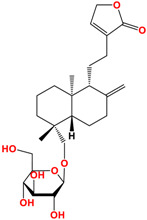 C_26_H_40_O_8_ MW: 480.59 g/mol
14-deoxyandrographolide	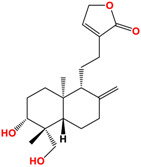 C_20_H_30_O_4_ MW: 334.45 g/mol
14-deoxy-11,12-didehydroandrographolide	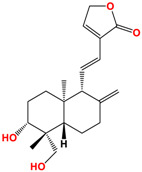 C_20_H_28_O_4_ MW: 332.43 g/mol
7,8-dimethoxy-2′-hydroxy-5-O-β-glucopyranosyloxyflavone	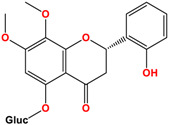 C_23_H_25_O_11_ MW: 477.14 g/mol
Luteolin	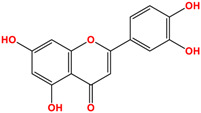 C_15_H_10_O_6_ MW: 286.23 g/mol

**Table 3:** Structure of main compounds found in *Curcuma longa* extracts and tetrahydrocurcumin.

Name	Structure and Details
Curcumin	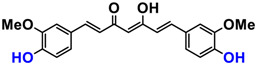 C_21_H_20_O_6_ MW: 368.38 g/mol
Demethoxy-curcumin	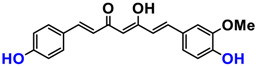 C_20_H_18_O_5_ MW: 338.35 g/mol
Bis-demethoxy-curcumin	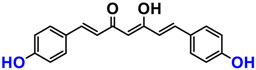 C_14_H_16_O_4_ MW: 248.27 g/mol
Tetrahydrocurcumin (THC)	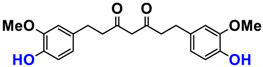 C_21_H_24_O_6_ MW: 372.41 g/mol

**Table 4:** Summary of the main properties and formulations of compounds found in *Camellia sinensis*, *Andrographis paniculata*, and *Curcuma longa*.

Plants	Compounds, Uses, and Methods	Results	Refs
*Camellia sinensis*	Biological activities	Neurological and obesity disorders, cardiovascular pathologies, antioxidant, anti-microbial, anti-inflammatory, and anti-carcinogenic activities. Regulation of Nrf2 signaling pathway and stimulation of the NF-kB and MAPK pathways.	[24,25,26,27,28,29,30,31,32,33,34,35]
	Extract of dried leaves	Main compounds: catechins, polyphenols, or flavan-3-ols.	[64,65]
		EGCG (65% of total catechins) depending on drying conditions and extraction process.	[65,66]
	Extraction of Catechins with polar organic solvents OR	Good extraction but unusable for cosmetics.	[67]
	Deep Eutectic solvents, for example: BGG-4 composed of betaine, glycerol, and D-(+)-glucose in the following proportions 4/20/1	Compatible with the use of extracts in cosmetics or pharmaceutical formulations.	
	Pharmacomodulations to increase the stability and bioavailability	Alkylated gallate esters, which retain very good antioxidant power and better bioavailability but have shown cytotoxicity in rats.	[28,65]
	Increased skin penetration with various formulations: chitosan microparticles (<5 mm) loaded with green tea extracts	Better skin permeability for non-galloylated catechins such as EGC and EC. Lower permeability for galloylated analogues of EGCG and ECG.	[70]
	EGCG reduces melanin secretion	Skin-lightening effect.	[71]
	Methylated form of EGCG: (3″ Me-EGCG)	Increase expression of heme oxygenase 1 (HO-1) protecting keratinocytes and antioxidant properties.	[72]
	Cosmetic formulation:		
	Emulsion, encapsulation, micro and nanoparticles	For better penetration of all three skin layers.	[73]
	Use of ethosomes	To protect EGCG from environmental aggressions.	[74,75]
	Similar study on niosome-loaded EGCG	To improve skin penetration and protect encapsulated molecules.	[22]
	Lipid nanoparticles to load EGCG	For cutaneous and transdermal drug delivery.	[77]
	Photosensitivity of EGCG	Synergistic effect of other anti-oxidant vitamins E and/or C, BHT, α-lipoic acid.	[78]
	Instability as a function of Ph values and UV irradiation	Decreased performance.	[79,80]
*Andrographis panilculata*	Biological activities	Anti-inflammatory, antipyretic, hepatoprotective, anti-thrombotic, immunostimulant, anti-viral, antioxidant, and anti-cancer.	[11,36,37,38,39,40,41,42,43,44,45,46,47,48,49,50,51,52,53]
		Regulation of the Nrf2 signaling pathway.	[42,86,87,94,95,96,97,98,99]
		Decreased ROS uptake or TNF-α expression under pro-oxidative and pro-inflammatory conditions.	[88,89,90,91]
		Decrease type I procollagen in HDFa	[91]
		Andrographolide can restore SOD and CAT activity in cells under oxidative stress.	[92,93]
	Extraction with polar organic solvents such as acetone, alcohols, and ethers but insolubility of andrographolide in water.	27 compounds listed including 4 main ent-labadanes.	[36,81,83]
	Methanolic extract	Presence of 0.87% andrographolide.	[39,53]
	Organ where storage takes place	Stored mainly in the kidneys and liver but also crosses the brain barrier.	[82]
	Microparticles, microemulsions, and nanocarriers	Formulation increases the bioavailability of andrographolide by 241% using nanoparticles compared to suspension.	[83,84]
	Nanoemulsions	Formulation of andrographolide for skin cancer with good distribution.	[102]
		Gel formulation for epidermal melasma with good tolerance.	[103]
*Curcuma longa*	Biological activities	Rheumatoid arthritis, anti-inflammatory, antioxidant, anticarcinogenic, chemopreventive, and anti-tumor.	[56,57,105,106,107]
	Instability	In biological media in vitro and in vivo.	[110]
	Dry skin problem	Curcumin treats skin disorders as dermatitis and itching.	[59,60,61,62]
	In vivo reduction of curcumin to tetrahydrocurcuminoids (THC)	Both have antioxidant or skin-lightening properties, depending on the dose. Curcumin and THC have different mechanisms of action.	[62]

## Data Availability

Not applicable.
